# Habit and Help—Experiences of Technology Use During the COVID-19 Pandemic: Interview Study Among Older Adults

**DOI:** 10.2196/58242

**Published:** 2024-10-18

**Authors:** Tina R Kilaberia, Yuanyuan Hu, Janice F Bell

**Affiliations:** 1 Silver School of Social Work New York University New York, NY United States; 2 School of Social Work University of Minnesota, Twin Cities St. Paul, MN United States; 3 Betty Irene Moore School of Nursing University of California, Davis Health Sacramento, CA United States

**Keywords:** pandemic, older people, technology habit, subjective experience, acceptance of technology

## Abstract

**Background:**

The COVID-19 pandemic compelled older adults to engage with technology to a greater extent given emergent public health observance and home-sheltering restrictions in the United States. This study examined subjective experiences of technology use among older adults as a result of unforeseen and widespread public health guidance catalyzing their use of technology differently, more often, or in new ways.

**Objective:**

This study aimed to explore whether older adults scoring higher on the Unified Theory of Acceptance and Use of Technology questionnaire fared better in aspects of technology use, and reported better subjective experiences, in comparison with those scoring lower.

**Methods:**

A qualitative study using prevalence and thematic analyses of data from 18 older adults (mean age 79 years) in 2 groups: 9 scoring higher and 9 scoring lower on the Unified Theory of Acceptance and Use of Technology questionnaire.

**Results:**

Older adults were fairly competent technology users across both higher- and lower-scoring groups. The higher-scoring group noted greater use of technology in terms of telehealth and getting groceries and household items. Cognitive difficulty was described only among the lower-scoring group; they used technology less to get groceries and household items and to obtain health information. Qualitative themes depict the role of habit in technology use, enthusiasm about technology buttressed by the protective role of technology, challenges in technology use, and getting help regardless of technology mastery.

**Conclusions:**

Whereas the pandemic compelled older adults to alter or increase technology use, it did not change their global outlook on technology use. Older adults’ prepandemic habits of technology use and available help influenced the degree to which they made use of technology during the COVID-19 pandemic.

## Introduction

Preventive public health measures in response to the COVID-19 pandemic, such as social distancing and sheltering in place, compelled older adults to rely on technology to a greater extent [1-3]. A study conducted in the United States found that 16%-26% of older adults increased remote contact with family or friends using cell phones, virtual meetings, and messaging applications [4]. Because mitigating the COVID-19 infection risk and seeking health services was important to many older adults during the pandemic [5-7], technology use was driven in large part by health-motivated reasons. Accordingly, many studies focused on older adults’ telehealth use and experiences, finding that 21.1% of older adults used telehealth, 4 times more than prepandemic [8]. For older adults with diabetes, technology was critical in meeting medical needs [9].

Many older people experienced barriers to telehealth use with the shift to this novel way of using technology to meet health care needs. Over 30% of older adults were not ready for video health visits [10]. In total, 82% of 873 homebound older adults surveyed in New York City required a caregiver’s assistance in completing a telehealth visit, with reported barriers including cognitive or sensory impairment and lack of access to caregiver assistance, which prevented 27% of participants from using telehealth technology [11]. Positively and significantly associated with telehealth use were the number of medical conditions, impairment in instrumental activities of daily living, owning technology devices, having a working computer or tablet, online use experiences, and new technology learning during the pandemic [8].

Studies highlighted disparities related to accessibility and use of telehealth services. Older people who lived in nonmetro areas, had no internet access, or did not have any experience using teleconferencing platforms were less likely to access telehealth; and being Hispanic and non-Hispanic Black with multiple comorbidities were statistically significant predictors for telehealth use [12]. Older adults with low English proficiency faced language barriers in independent living facilities whereas for participants with high English proficiency and a higher level of education, barriers included unfamiliarity with the technology and difficulties connecting to telehealth platforms [13].

Aside from telehealth, older adults used technology during the pandemic for leisure and entertainment, social connectedness, and education and information seeking [1,3,5,14]. Facilitators of technology uptake were pursuing emotionally meaningful goals [15], perceiving usefulness of technology [16], and living with family or having technology help from family [17,18]. Barriers included limited interest in new technology training among those aged 80 years and older due to a perception of having less time left (to live) to make use of that training [2,15]; physical limitations, native language other than English, perceived high cost, lack of infrastructure [17]; culture and facilitating conditions [1]; and being male, having less than high school education, lower income, and self-reported fair or poor general health [2].

Studies note similar drivers of technology use by older people before the pandemic with regard to leisure and gaming [19], and social networking, online chatting, instant messaging, and video calls [20]. Facilitators of technology uptake by older people before the pandemic were likewise similar, and pertained to perceived usefulness, social benefits, and living with children [21]; better self-rated health, fewer chronic illnesses, higher subjective well-being, and fewer depressive symptoms [20]. Barriers to technology uptake pertained to limited access and low technological self-efficacy [22]; low technology literacy and physical challenges [23]. The purpose of prepandemic studies of technology use by older adults is to examine the role of technology in alleviating loneliness and social isolation [20,24], and to examine openness of older people toward emerging technologies to meet additional needs [25].

Less is known about technology experiences of older adults in subjective terms at a time of unforeseen and sudden-onset public health measures to mitigate the risks of COVID-19 pandemic, which made the use of technology less optional in comparison with before the pandemic. Many older adults had not planned or prepared to rely on technology more than they already had, and in ways they had not before, yet home-sheltering forced them to increase technology use at the same time that their caregivers who ordinarily help them, including helping with technology [17,18] were themselves experiencing multiple stressors [26]. The current study adds knowledge to this context by examining the subjective experiences in older people’s technology use to meet their needs during the pandemic, which catalyzed their technology use and compelled them to use it differently, more often, or in new ways.

## Methods

### Study Design

This study converged qualitative semistructured in-depth interviews about technology use during the pandemic with descriptive data garnered from the Unified Theory of Acceptance and Use of Technology (UTAUT) questionnaire [27]. Both qualitative and quantitative descriptive data were collected concurrently, with qualitative questions preceding the survey responses [28]. Whereas qualitative interviews elicited subjective perspectives, survey questions elicited descriptive information that allowed subdividing participants into 2 comparison groups as described below.

### Recruitment

The study sample was recruited from September 2020 through July 2021, during which time there were active public health guidance and home-sheltering mandates across many states in the United States. Flyers were distributed electronically to social service providers, in the national family caregiver alliance research registry targeting stakeholders and service providers, and in 2 congregate housing and care communities, 1 in the Midwest and 1 on the West Coast in the United States. In addition, we employed a public health diversity expert to increase participation by older adults from minoritized groups.

The sample’s mean age was 79 years with 7 (39%) older adults being at least 80 years old or older. Twelve (67%) were White, and as many reported being female, 8 (44%) lived either in congregate housing or with family, with the rest being community-dwelling. Two older adults described themselves as employed part-time and not working outside the home for pay. All others considered themselves retired.

### Data Collection

Both qualitative and survey data were collected over Zoom [29] and telephone. First, qualitative questions elicited information about experiences of technology use before the pandemic, changes in technology use resulting from the pandemic, types of and purposes for technology use, and managing the challenges of technology use at the same time that older people managed the pandemic with technology. Subsequently, 5 sections of the UTAUT questionnaire [27] on attitude, facilitating conditions, self-efficacy, anxiety, and behavioral intention were administered, amounting to 19 UTAUT survey questions, in total, asked of every participant.

### Data Analysis

#### Prevalence Comparison

Given the analytic interest in comparing technology use patterns between those reporting greater and lesser acceptance and use of technology, 18 older adults were split into 2 groups, those scoring above the median (n=9) and below the median (n=9) based on the average score of the 5 sections of UTAUT as described above [27]. The coded qualitative dataset was split accordingly, to compare the prevalence in aspects of technology use across higher and lower-scoring groups. Our rationale for using 2 comparison groups was guided by an expectation that higher-scoring older adults would fare better in aspects of technology use, and report better (less challenging) experiences, in comparison with those scoring lower.

#### Qualitative Thematic Analysis

Qualitative interviews lasted 42 (range 21-74) minutes on average, were transcribed verbatim by a professional transcriptionist and deidentified. Qualitative thematic analysis of interview data proceeded in phases following Braun and Clarke’s method [30]. In phase 1, all authors familiarized themselves with the data by reading transcripts. In phase 2, data were coded independently and inductively by all authors, developing initial codes. In phase 3, individually developed codes were compared, contrasted, and refined in 8 iterations of coding over the course of 5 months, keeping a record of memos in addition. Discussion of potential themes occurred at this stage. In phase 4, codes were differentiated by themes and subthemes, for example, enthusiasm and reluctance toward technology use were reflected as themes; and the “protective role of technology” as a subtheme of “enthusiasm about technology.” Phase 5 final themes [30] are a result of this coding process, with the final themes labeled as follows: (a) the role of habit in technology use, (b) enthusiasm about technology buttressed by (b1) the protective role of technology, (c) challenges in technology use, and (d) getting help regardless of technology mastery. For quotes presented below, Krippendorff α [31] was computed for intercoder reliability based on the independent rating of the 3 authors.

NVivo Pro (Lumivero) [32] was used to manage and code qualitative data whereas survey data were entered in Microsoft Excel and summarized with descriptive statistics. Stata (StataCorp) [33] was used to calculate Krippendorff α.

### Reflexivity

The first author had a prolonged immersive experience observing older adults’ lives in a congregate setting some time before undertaking this study [34,35], with a focus on social relationships and social isolation [36,37]. The first author undertook this present study to explore the role of technology in social connectedness among older adults at a time when, in observance of the COVID-19–related mitigation strategies of home-sheltering and social distancing across the United States, their social ties were disrupted. At the time of data analysis and drafting the manuscript, coauthors were from diverse academic backgrounds representing social work, public health, and nursing, and reflecting expertise in health and mental health services research.

### Ethical Considerations

The University of California, Davis Health Institutional Review Board approved the study before initiation (approval number 1646206-1). Following telephone eligibility screening, participants provided verbal informed consent to the first author and received a US $25 gift card each for participation.

## Results

### Description of the Study Sample

As shown in Table 1, higher-scoring participants (above the median 4.09) were primarily female (8/9, 89%), younger (mean age 79 years), highly educated with either college or graduate degrees (7/9, 78%) with more than half living in congregate housing (5/9, 56%). By contrast, more than half of the lower-scoring participants were male (5/9, 56%) and slightly older (mean age 80 years); a lower percentage had college or graduate degrees (6/9, 67%) with one-third in each category of some college, college graduate, and graduate degree; and a higher percentage were community-dwelling (6/9, 67%). In the 2 analysis groups, equal proportions were married (5 in each group or 56%) and reported physical health challenges (6 in each group or 67%).

**Table 1:** Description of the study sample across higher- and lower-scoring older adults on Unified Theory of Acceptance and Use of Technology (UTAUT) questionnaire.

Characteristics			UTAUT questionnaire (Median=4.09, N=18)		
			Above median (n=9)		Below median (n=9)
Age (years), mean			78.67		79.89
**Gender, n (%)**					
	Male	1 (11)		5 (56)	
	Female	8 (89)		4 (44)	
**Race and ethnicity, n (%)**					
	White	6 (67)		6 (67)	
	Black or African American	3 (33)		2 (22)	
	White, Other	0 (0)		1 (11)	
**Marital status, n (%)**					
	Widowed	2 (22)		1 (11)	
	Married	5 (56)		5 (56)	
	Divorced	2 (22)		3 (33)	
**Education level, n (%)**					
	High school diploma/GED^a^	1 (11)		0 (0)	
	High school diploma /GED, professional certificates	1 (11)		0 (0)	
	Some college	0 (0)		3 (33)	
	College graduate	3 (33)		2 (22)	
	College graduate, some graduate school	0 (0)		1 (11)	
	Graduate degree	4 (44)		3 (33)	
**Employment status, n (%)**					
	Consider myself retired	7 (78)		9 (100)	
	Employed part-time	1 (11)		0 (0)	
	Not working outside the home for pay	1 (11)		0 (0)	
**Physical health challenges, n (%)**					
	Yes	6 (67)		6 (67)	
	No	3 (33)		3 (33)	
**Annual income** **(in thousands; US $), n (%)**					
	<25	0 (0)		1 (11)	
	<25, Savings	0 (0)		1 (11)	
	25-49	4 (44)		4 (44)	
	50-99	3 (33)		3 (33)	
	50-99, Savings	1 (11)		0 (0)	
	≥100	1 (11)		0 (0)	
**Living arrangement, n (%)**					
	Congregate housing	5 (56)		2 (22)	
	Community-dwelling	4 (44)		6 (67)	
	With caregiver’s family	0 (0)		1 (11)	

^a^GED: General Educational Development.

### Prevalence Comparison Results

Laptop computers were more common among those above the median UTAUT score (5/9, 56% vs 1/9, 11%), and smartphones were equally common in both groups (3/9, 33%). Although landline phones are not currently always considered to be technology, 1 person (11%) in each group identified such phones as go-to technology (technology relied on regularly). Desktop computers (3/9, 33%) and iPads (1/9, 11%) were identified as go-to technology only in the group with lower UTAUT scores.

All older adults with higher UTAUT scores had used technology before the pandemic in comparison to 7 (78%) of those scoring lower. One-third (33%) in each group reported that their use of technology changed (they used a different type of technology in addition to the ones used ordinarily). Of those scoring higher, 5 (56%) had increased their use of technology due to the pandemic in comparison with 3 (33%) among those scoring lower. An equal proportion used technology to stay in touch with family (6/9, 67%). A higher percentage of older adults used telehealth or MyChart for health-related information in the group with higher UTAUT scores as compared with the group with lower scores (6/9, 67% vs 4/9, 44%). Almost all of those with higher scores used technology to get groceries and household items (8/9, 89%) in comparison with 4 (44%) in the lower-scoring group. Cognitive difficulty was described only in the lower-scoring group (4/9, 44%).

### Qualitative Thematic Analysis Results

The thematic analysis furthered these findings in subjective terms, and depicted (a) the role of habit in technology use, (b) enthusiasm about technology buttressed by (b1) the protective role of technology, (c) challenges in technology use, and (d) getting help regardless of technology mastery. Krippendorff α equaled 0.97 for intercoder reliability among 3 coders. The themes are elaborated below with illustrative quotes.

#### Theme 1: The Role of Habit in Technology Use

Twelve (67%) older adults had engaged with technology during the pandemic as they had done before, in typical or habitual ways: if they were avid technology users before the pandemic, they remained so during the pandemic. Conversely, those who preferred in-person contact, as opposed to interactions through technology, continued doing so, noting observance of public health and safety guidance when needed:

I haven’t done any [online shopping] since [the pandemic started]. I just prefer to see and feel and shop. I never bought anything from a catalog. […] it doesn’t bother anybody else [laughs], I know. But it bothers me.

Participant 1

By contrast, some older adults had shopped almost entirely online before the pandemic and continued doing so during the pandemic:

I’ve been an online shopper, I’m thinking, for probably maybe 30 years. […] I haven’t been in a department store, or a shoe store, or anything like that for a long, long time.

Participant 2

One older adult had maintained learned knowledge about using technology over a number of years, and as long as the learned routine could be carried over during the pandemic, she made use of it:

I'm not very comfortable with computers. I've used them for years, but you know, I have used them in fairly limited circumstances that had turned into routines that I didn't have to spend a lot of time guessing about how to do something.

Participant 3

#### Theme 2: Enthusiasm About Technology: “Lots of Ways to Do Everything”

Thirteen (72%) older adults expressed enthusiasm about using technology during the COVID-19 pandemic. It encompassed a range of situations in which they used technology, as examples, for telehealth, communication, entertainment, and spending time socially with others:

The couple times when I needed to see my doctor, we did a Zoom. [...] I talk to my cardiologist because he wants to see me and assess me. So yes, I’ve used telehealth a lot. I like it, but it has its limitations.

Participant 4

I have an iPhone. And an Apple Watch. I use the computer to communicate. The Apple Watch makes sure I notice a call on my phone. Put the three things together and it’s pretty communicative really.

Participant 5

Opera Company is doing an opera for the next three weekends and they’re doing it in 3D and with virtual reality. I’ve got two tickets. They sent me two pairs of 3D glasses. I’ve got a friend who’s got a couple of virtual reality headsets. So, I’ll plug my computer into my TV and the code they sent me and will be able to see the opera then in 3D and virtual reality! So, they’ve found a way to monetize the art and a way to present it, that we can stay home and safe and still enjoy the performance!

Participant 6

Of 13 older adults, 6 (46%) expressing enthusiasm referred to technology as most protective during the pandemic, reflecting subtheme 2A: Protective Role of Technology. Most meaningful to them was the use of online meeting technologies to keep connected and visit socially with others:

“[Before the pandemic], I had never used Zoom or GoTo Meeting” (video web conferencing and online meeting software)

Participant 7

Older adults described being aided by technology in remaining connected with others and feeling less isolated:

It’s imperative. Anyone that does not have either a smartphone, or a laptop, or a computer, I’m not even sure how… I couldn’t even begin to evaluate their existence.

Participant 8

The phone, I think is the one thing I really would not want to be without when you're going through some type of illness or pandemic or whatever. Because that can keep you in contact, just by phone to even talk for a few minutes. Makes a big difference.

Participant 9

Some reported switching to shopping online in consideration of public health safety guidance as protective because their needs could still be met even if not in person:

“I didn't clothes shop or grocery shop online” (prior to the pandemic).

Participant 10

#### Theme 3: Challenges in Technology Use: “I Just Get Through It.”

A smaller group of older adults (5/18, 28%) reported some reluctance in using technology, and even delayed or foregone health care, due to challenges navigating technology or its being impersonal.

I think that's been kind of an irritation [about telehealth], not being able to get a doctor's appointment without sitting and talking to a screen, which I'd rather talk in person [...]. So that's been kind of irritating. [...] Right now my left shoulder hurts like hell, they want me to go to the doctor but I don't want to go to no screen. That's an irritation.

Participant 11

Challenges ranged from lack of knowledge, being uncomfortable or frustrated, and living in a rural location with limited capacity to fully engage technology:

Sometimes this phone drives me completely nuts. I could snatch it out by the wires, but unfortunately, you know, my heart monitor's hooked up to it too. So, if it wasn't for that, I really would. I would snatch this thing out and throw it out in the river!

Participant 12

It’s one of the disadvantages of living where we do [rurally] is that sometimes our technology doesn’t work. Sometimes, our power goes out, but we deal with it. We just live with it. That’s the way it is.

Participant 13

#### Theme 4: Getting Help With Technology Regardless of Technology Mastery

Almost all older adults (17/18, 94%) reported needing help with technology. Most older adults referred to family and friends as sources of help, some identified paid services, and those living in congregate housing identified additional built-in supports for technology help:

My son, my daughter, and my grandkids [help me]. I have a tablet and telephone. I got a new telephone [...] the end of last year. And [...] my son had to come over and show me how to use it.

Participant 14

I called people and stayed on the telephone for hours to get it straight. Sometimes... Like, I just switched over to [provider X] for my TV, and I couldn’t get that straight, and I had to call the technician to come back out here. I said, “You have to come back out here and break it down like I’m five years old because I’m not getting this.” So they sent a technician out here.

Participant 15

They do have somebody here [in congregate housing] that will—I think it’s for two hours, one day a week, you can schedule an appointment with that person to help with technology.

Participant 16

## Discussion

### Principal Findings: Habit and Help in Technology Use

This study examined older adults’ experiences of technology use by converging qualitative data with the UTAUT questionnaire [27]. Although we categorized older adults according to their UTAUT scores, both higher- and lower-scoring groups had relatively high scores, suggesting good technology access and competence in use overall. Studies reflecting technology use by older people both before [22,23] and during the pandemic [2,17] have reported that older people experience challenges due to limitations in technology literacy and health status. By contrast, this study showed a generally high mastery of various technology types used by older adults during the COVID-19 pandemic. At the same time, we found nuanced differences between older adults scoring higher and lower on UTAUT in their preference for devices, use of technology to meet needs, and cognitive capacity. Further research in larger samples could corroborate these findings, and help determine if subtle differences in technology access and use across both groups in the sample might indicate the need for different levels or types of support, particularly among older adults with mild cognitive impairment.

On the whole, our findings suggest that the pandemic did not necessarily compel older people to learn or commit to new technologies even if their technology use increased during the pandemic. Long-standing habits may influence engagement with technology even when engagement is catalyzed by an emergent circumstance such as the pandemic. In this study, long-standing habits influenced preferences toward desktop computers, landline phones, and in-person shopping, reflecting decades’ worth of technology mastery in a historical sense. Older adults in our sample used technology much in the same way as before qualitatively. Such habitual engagement with technology may in turn affect reluctance to adopt new or different technologies. Studies showed that older people’s openness varied toward emerging technologies with under half (48%) being open to internet-connected cameras for home monitoring and few (15%) being open to virtual reality [25]. Less than half (43%) of people who were offered telehealth services by their medical providers used such services [12]. Habit, being used to certain technology or using it for specific needs and not for other needs, may play a part in technology use among older adults even under compelling circumstances such as the pandemic. The sudden need due to the pandemic and related public health guidance may have pushed older adults to alter or increase technology use. However, it did not change their overall outlook on technology use. Older adults’ previous habits in both using technology in the same way and using same technology used historically, influenced the degree to which they made use of technology during the COVID-19 pandemic.

Despite all older adults scoring quite high on technology access and use, both groups reported similar needs for technology support. Most often, this support came from family members. This substantiates findings that both before [21] and during [11,17,18] the pandemic, living with family or having technology help from family or a paid caregiver is helpful to older adults’ uptake and engagement with technology. We contrast this with the fact that more than half of the higher-scoring participants in our study lived in congregate settings, where they may have benefitted from on-site technology support or embedded friendship ties that they could draw on for technology support. The need for help noted across both groups suggests that despite competence, easy access to technology support may help engagement with technology and enhance greater social connectedness, especially for community or rurally dwelling older adults who may not have such support readily available.

### Changed Use of Technology: Telehealth and Online Meeting Platforms

Telehealth and online meeting platforms (such as Zoom) were 2 areas in which older adults engaged with technology in ways in which they had not before the pandemic: participants scoring higher on the UTAUT questionnaire used technology to a greater extent for telehealth than those scoring lower even as the proportion of older adults reporting physical health challenges was the same in both groups. This relates to a previous finding of a 4-fold increase in the use of telehealth by older adults after the start of the pandemic [8], and suggests that greater technology mastery may help in telehealth use. We note that the use of telehealth and online meeting platforms, described as a means to maintain social connection, was also true for younger segments of the population during the pandemic [38]. Likewise, the willingness to adopt these new platforms, in our sample of older adults already comfortable with technology, and with access to technology support through family and other means, was likely driven by the perceived benefit to quality of life resulting from engagement in telehealth and online social connection visits. This is consistent with previous studies finding that motivation from meaningful goals and perceived value [15,16,39] facilitate technology engagement.

### Increased Use of Technology for Household Needs

Participants with higher UTAUT scores increased their technology use for household needs to a greater extent than their counterparts with lower scores in this study. Given that our higher-scoring sample was predominantly female with graduate education, our study lends some support to the finding that being male and having lower than high school education was a barrier to learning a new technology to go online during COVID-19 pandemic [2]. Although our sample was small, we note additionally with regard to the digital divide that 2 of 3 older adults living in rural locations highlighted challenges of access to technology, a finding reflected in previous studies [12,40]. In addition, cognitive difficulty, noted in this study only in the lower scoring group, and juxtaposed with their comparatively lower use of technology to get groceries and household items and to obtain health information, suggests the need for various types of help and support among older people. Technology help may be insufficient for older adults with cognitive impairment who may not remember how to navigate technology despite being helped, and may require qualitatively different types of support in addition.

### Limitations

Enabled by the deliberate recruitment of minoritized older adults, a strength of this study is that 28% of the sample represented diverse groups, with 1 identifying as Other aside from White, non-Hispanic. We also included participants from more than 1 part of the country in the United States adding to the geographic diversity. At the same time, as a pilot study, the sample size is small (18 older adults), representing older people more educated (72% with college or graduate degree) and economically better off (83% reporting income between US $25,000 and US $99,999). These attributes may have affected the willingness and ability to participate in a study using the Zoom technology, and may reflect the generally high technology access and use scores in the sample. Future research is recommended in samples with lower income and education to corroborate our results.

### Conclusions

The COVID-19 pandemic catalyzed older adults’ technology use as a result of public health guidance and home-sheltering associated with the pandemic. However, older adults in this study with relatively high technology access and use did not report substantial changes in their technology use in meeting their needs. In fact, previous technology habits, feeling protected (adding to enthusiasm) through the use of technology, and the availability of technology support influenced the degree to which older people made use of technology in the context of the pandemic. We found that older adults in this study were willing to adopt new technology including telehealth and online meeting platforms, perhaps because of their perceived benefits and potential to improve quality of life. Further research is recommended to corroborate the subtle differences we found in the socio-demographic characteristics, cognitive capacity, technology preferences, and technology use during the pandemic between the 2 groups of older adults at both higher and lower ends of technology access and use scores in the sample. Different types of support may be required, even by older adults who have access and are comfortable using technology, especially in the context of cognitive decline.

## Abbreviations

UTAUT: Unified Theory of Acceptance and Use of Technology

## Data Availability

The datasets generated and analyzed during this study are not publicly available due to the qualitative nature of the study and associated ethics review restrictions, but are available from the corresponding author on reasonable request.

## References

[1] Elimelech OC, Ferrante S, Josman N, Meyer S, Lunardini F, Gómez-Raja J, Galán C, Cáceres P, Sciama P, Gros M, Vurro C, Rosenblum S (2022). Technology use characteristics among older adults during the COVID-19 pandemic: a cross-cultural survey. Technol Soc.

[2] Li W, Ornstein KA, Li Y, Liu B (2021). Barriers to learning a new technology to go online among older adults during the COVID-19 pandemic. J Am Geriatr Soc.

[3] Sixsmith A, Horst BR, Simeonov D, Mihailidis A (2022). Older people's use of digital technology during the COVID-19 pandemic. Bull Sci Technol Soc.

[4] Hawkley LC, Finch LE, Kotwal AA, Waite LJ (2021). Can remote social contact replace in-person contact to protect mental health among older adults?. J Am Geriatr Soc.

[5] Chen AT, Ge S, Cho S, Teng AK, Chu F, Demiris G, Zaslavsky O (2021). Reactions to COVID-19, information and technology use, and social connectedness among older adults with pre-frailty and frailty. Geriatr Nurs.

[6] de Feijter M, Kocevska D, Blanken TF, van der Velpen IF, Ikram MA, Luik AI (2022). The network of psychosocial health in middle-aged and older adults during the first COVID-19 lockdown. Soc Psychiatry Psychiatr Epidemiol.

[7] Kilaberia TR, Hu Y, Ratner ER, Bell JF (2024). Comparing social isolation in older adults with and without physical health challenges during COVID-19: church and church friends matter. INQUIRY: The Journal of Health Care Organization, Provision, and Financing.

[8] Choi NG, DiNitto DM, Marti CN, Choi BY (2022). Telehealth use among older adults during COVID-19: associations with sociodemographic and health characteristics, technology device ownership, and technology learning. J Appl Gerontol.

[9] Toschi E, Slyne C, Weinger K, Sy S, Sifre K, Michals A, Davis D, Dewar R, Atakov-Castillo A, Haque S, Cummings S, Brown S, Munshi M (2022). Use of telecommunication and diabetes-related technologies in older adults with type 1 diabetes during a time of sudden isolation: mixed methods study. JMIR Diabetes.

[10] Perry L, Scheerens C, Greene M, Shi Y, Onion Z, Bayudan E, Stern RJ, Gilissen J, Chodos AH (2023). Unmet health-related needs of community-dwelling older adults during COVID-19 lockdown in a diverse urban cohort. J Am Geriatr Soc.

[11] Kalicki AV, Moody KA, Franzosa E, Gliatto PM, Ornstein KA (2021). Barriers to telehealth access among homebound older adults. J Am Geriatr Soc.

[12] Ng BP, Park C, Silverman CL, Eckhoff DO, Guest JC, Díaz DA (2022). Accessibility and utilisation of telehealth services among older adults during COVID-19 pandemic in the United States. Health Soc Care Community.

[13] Mao A, Tam L, Xu A, Osborn K, Sheffrin M, Gould C, Schillinger E, Martin M, Mesias M (2022). Barriers to telemedicine video visits for older adults in independent living facilities: mixed methods cross-sectional needs assessment. JMIR Aging.

[14] Kim S, Yao W, Du X (2022). Exploring older adults' adoption and use of a tablet computer during COVID-19: longitudinal pualitative study. JMIR Aging.

[15] Bardach SH, Rhodus EK, Parsons K, Gibson AK (2021). Older adults' adaptations to the call for social distancing and use of technology: insights from socioemotional selectivity theory and lived experiences. J Appl Gerontol.

[16] Ma Q, Chan AHS, Teh PL (2021). Insights into older adults’ technology acceptance through meta-analysis. International Journal of Human–Computer Interaction.

[17] Keohane S, Swarbrick C, Helal S, Gao Q, Zhou J (2022). Barriers and facilitators to technology among older adults during COVID-19 pandemic: a systematic review using thematic analysis. Human Aspects of IT for the Aged Population: Design, Interaction and Technology Acceptance.

[18] Lee L, Maher ML (2021). Factors affecting the initial engagement of older adults in the use of interactive technology. Int J Environ Res Public Health.

[19] Boot WR, Andringa R, Harrell ER, Dieciuc MA, Roque NA (2020). Older adults and video gaming for leisure: lessons from the center for research and education on aging and technology enhancement (CREATE). Gerontechnology.

[20] Chopik WJ (2016). The benefits of social technology use among older adults are mediated by reduced loneliness. Cyberpsychol Behav Soc Netw.

[21] Liu D, Liu A, Tu W (2020). The acceptance behavior of new media entertainment among older adults: living arrangement as a mediator. Int J Aging Hum Dev.

[22] Tsai HS, Shillair R, Cotten SR, Winstead V, Yost E (2015). Getting grandma online: are tablets the answer for increasing digital inclusion for older adults in the U.S.?. Educ Gerontol.

[23] Wang S, Bolling K, Mao W, Reichstadt J, Jeste D, Kim HC, Nebeker C (2019). Technology to support aging in place: older adults' perspectives. Healthcare (Basel).

[24] Czaja SJ, Boot WR, Charness N, Rogers WA, Sharit J (2018). Improving social support for older adults through technology: findings from the PRISM randomized controlled trial. Gerontologist.

[25] Kadylak T, Cotten SR (2020). United States older adults’ willingness to use emerging technologies. Information, Communication & Society.

[26] Kilaberia T, Bell J, Bettega K, Mongoven J, Kelly K, Young H (2020). Impact of the COVID-19 pandemic on family caregivers. Innovation in Aging.

[27] Venkatesh V, Morris MG, Davis GB, Davis FD (2003). User acceptance of information technology: toward a unified view. MIS Quarterly.

[28] Padgett DK (2016). Qualitative Methods in Social Work Research.

[29] Zoom Video Communications (2020). Zoom workplace. Computer Software.

[30] Braun V, Clarke V (2006). Using thematic analysis in psychology. Qualitative Research in Psychology.

[31] Hayes AF, Krippendorff K (2007). Answering the call for a standard reliability measure for coding data. Communication Methods and Measures.

[32] Lumivero (2022). What's new: release 1.7.1.

[33] StataCorp (2021). Stata Statistical Software: Release 17. Computer Software.

[34] Kilaberia R, Ratner E (2018). Live-in "strangers": an experiential account of gerontology educational immersion in senior housing. Gerontol Geriatr Educ.

[35] (2017). Housing seniors with students.

[36] Kilaberia TR (2021). Negotiating social diversity in residential care for older persons. Journal of Contemporary Ethnography.

[37] Kilaberia TR, Chang E, Padgett D, Lachs M, Rosen AE (2024). "What does 'age-friendly' mean to you?" The role of resident-to-resident microaggressions in a retirement and assisted living community. The Gerontologist.

[38] David ME, Roberts JA (2021). Smartphone use during the COVID-19 pandemic: social versus physical distancing. Int J Environ Res Public Health.

[39] Moxley J, Sharit J, Czaja SJ (2022). The factors influencing older adults' decisions surrounding adoption of technology: quantitative experimental study. JMIR Aging.

[40] Young HM, Bell JF, Tonkikh O, Kilaberia TR, Whitney RL, Mongoven JM, Link BM, Kelly K (2022). Implementation of a statewide web-based caregiver resource information system (CareNav): mixed methods study. JMIR Form Res.

